# Risk of Thyroxine-Treated Autoimmune Thyroid Disease Associated With Disease Onset in Patients With Rheumatoid Arthritis

**DOI:** 10.1001/jamanetworkopen.2018.3567

**Published:** 2018-10-19

**Authors:** Kristin Waldenlind, Saedis Saevarsdottir, Camilla Bengtsson, Johan Askling

**Affiliations:** 1 Department of Medicine, Solna, Karolinska Institutet, Karolinska University Hospital, Stockholm, Sweden; 2 Institute of Environmental Medicine, Karolinska Institutet, Stockholm, Sweden

## Abstract

**Importance:**

Autoimmune thyroid disease ([AITD] including hypothyroidism and hyperthyroidism) is the most common organ-specific autoimmune disorder and is more prevalent among patients with rheumatoid arthritis (RA). Real-world studies on when and how this increased risk of AITD develops, in association with the time before or after the onset of RA, are lacking.

**Objective:**

To estimate the risk of thyroxine-treated AITD among patients with RA at different time points before and after the diagnosis of RA.

**Design, Setting, and Participants:**

A nationwide register-based case-control and cohort study was conducted between January 1, 2006, and June 30, 2013, with a maximum follow-up time of 7 years before and 8 years after diagnosis of RA. The study used the Swedish Rheumatology Quality Register and linkage to other nationwide registers to identify 8090 adults with new-onset RA and a random population-based sample of 80 782 referents matched by age, sex, and residential area. Statistical analysis was performed from July 1, 2015, to June 30, 2017.

**Exposures:**

Presence of AITD in the participants in the case-control design and RA in the participants in the cohort design.

**Main Outcomes and Measures:**

Prevalence and relative risk of incident AITD before (odds ratios) and after (hazard ratios) diagnosis of RA compared with the population as reference.

**Results:**

There were 8090 patients with RA (5529 women and 2561 men; mean [SD] age, 58.3 [15.2] years) and 80 782 population-based participants as reference who were identified. By the time of diagnosis of RA, the prevalence of AITD was 10.3% among the patients with RA (n = 832) vs 7.1% among the controls (5725 of 80 350) (odds ratio, 1.5; 95% CI, 1.4-1.7). This increased risk of AITD developed during the 5 years (range, 2-5 years) before diagnosis of RA (odds ratio, 1.5; 95% CI, 1.2-1.8) and peaked by the time of diagnosis of RA (range, 0-3 months before diagnosis of RA) (odds ratio, 5.3; 95% CI, 3.7-7.6). From diagnosis of RA and onward, the risk of developing AITD decreased (range, 2-5 years after diagnosis of RA) (hazard ratio, 0.7; 95% CI, 0.5-1.0).

**Conclusions and Relevance:**

Compared with the general population, Swedish patients with RA appear to have a higher prevalence of thyroxine-treated AITD at diagnosis of RA and an increased incidence of AITD during the 5-year period before diagnosis of RA. After diagnosis of RA, the risk of developing AITD is suggested to decrease below the expected rate. Besides temporal changes in diagnostic intensity, this pattern of risk raises the question whether AITD may influence the pathogenesis of RA (or vice versa) and, conversely, the question whether antirheumatic therapies may prevent AITD.

## Key Points

**Question:**

When does increased risk of autoimmune thyroid disease occur among patients with rheumatoid arthritis?

**Findings:**

In this Swedish case-control and cohort study, the prevalence of autoimmune thyroid disease at diagnosis of rheumatoid arthritis was 10.3%. The risk of new-onset autoimmune thyroid disease increased during the 5 years before diagnosis of rheumatoid arthritis, peaked at diagnosis, and then decreased 2 to 5 years after diagnosis of rheumatoid arthritis.

**Meaning:**

The increased occurrence of autoimmune thyroid disease among patients with rheumatoid arthritis appears to develop during the years before diagnosis of rheumatoid arthritis; further research is needed to understand if the reduced risk of autoimmune thyroid disease after diagnosis of rheumatoid arthritis may be associated with antirheumatic therapies.

## Teaser

This register-based case-control and cohort study estimates the risk of thyroxine-treated autoimmune thyroid disease among Swedish patients with rheumatoid arthritis at different time points before and after the diagnosis of rheumatoid arthritis.

## Introduction

Autoimmune thyroid disease (AITD) is the most common organ-specific autoimmune disease in the general population, with a prevalence of around 5%.^1^ Autoimmune thyroid disease can be subgrouped into hypothyroidism (mainly Hashimoto thyroiditis) and hyperthyroidism (Graves disease). Among patients with rheumatoid arthritis (RA), the reported prevalence of AITD is increased, although the reported levels of increase vary with study design, geographical region, and the definition of AITD.^2,3,4,5,6,7^ Variants in genes (eg, *PTPN22*, *CTLA 4*, and *HLA-DR*) involved in the regulation of T-cell response have been associated with both RA and AITD, suggesting that shared inheritance may be one factor linking the 2 conditions together. Whether there are other links between the 2 conditions, such as a causal effect of AITD on the onset of RA, shared environmental triggers, and/or disease-modifying antirheumatic drugs exerting a protective effect on the development of AITD, remains unclear.^1,8^ An assessment of the timing of the risk among patients with RA of developing AITD may provide important clues to the nature of their association and may provide useful information for clinicians evaluating, diagnosing, and treating the 2 conditions.

Most previous studies describing the association between AITD and RA have used cross-sectional designs, and they have reported an increased prevalence of RA among patients with AITD,^9,10,11^ an increased prevalence of AITD among patients with RA,^8,11^ or an increased prevalence of thyroid autoantibodies in patients with RA.^7,8,12,13,14^ Although they demonstrate the co-occurrence of RA and AITD, these studies do not provide information on the risk of developing these conditions over time. Two studies of incident RA suggest that the increased occurrence of AITD may already exist by the time of the diagnosis of RA, raising the question of when this increased risk develops.^6,15^ Little is known about the risk of AITD after RA diagnosis.

Therefore, the objectives of our study were to assess the relative risk of thyroxine-treated AITD among patients with RA at various time points before and after diagnosis of RA compared with the general population and, specifically, to assess whether the risk was substantially higher among different subgroups of patients with RA (seropositive vs seronegative) and AITD (hyperthyroidism vs hypothyroidism).

## Methods

### Setting and Data Sources

Swedish residents (approximately 10 million) have access to a publicly funded health care system. Most Swedish patients with RA are observed and treated by rheumatologists working in public hospital–based care. The Swedish personal identification number is unique to every resident and allows linkage between different nationwide health care registers, with essentially no losses to follow-up. The linkage and study were approved by the Stockholm Regional Ethics Committee (DNR 2009/2005-31/3). Informed consent from study participants is not required for register-based studies, according to Swedish law. This study followed the Strengthening the Reporting of Observational Studies in Epidemiology (STROBE) reporting guideline.

The Swedish Rheumatology Quality Register (SRQ) is a nationwide, clinically integrated register of patients with RA operated by the Swedish Society for Rheumatology since 1996. In the SRQ, early RA is defined as patients older than 18 years who fulfill the American College of Rheumatology criteria for RA^16^ or the American College of Rheumatology/European League Against Rheumatism classification criteria,^17^ with a reported symptom duration of less than 12 months. Information about race/ethnicity is not registered in the SRQ.

The Swedish Prescribed Drug Register (PDR) contains information on dispensations of prescribed drugs in Sweden since 2005. The coverage is close to 100%. A Swedish prescription is valid for 1 year, and a typical filling for medications for chronic conditions should cover 3 months’ use. The Swedish Cancer Register is nationwide and mandatory for all clinicians, resulting in almost complete coverage. The Swedish National Patient Register (NPR) includes diagnosis codes from hospital discharges (since 1964) and from outpatient visits in nonprimary outpatient care (since 2001). Diagnoses are coded according to the *International Classification of Diseases* (*ICD*), *Seventh to Ninth Revisions*, and the *International Statistical Classification of Diseases and Related Health Problems, Tenth Revision*. Coverage is close to 100% for inpatient visits and approximately 80% for outpatient visits. The Swedish Population Register and Cause of Death Register contain virtually complete information about residential area, date of emigration, and date of death.

### Study Population

For the case-control study, we identified all patients registered in the SRQ with a diagnosis of early RA between January 1, 2006, and June 30, 2013 (n = 8326). Of the 8326 patients with RA, 236 were excluded because they did not fulfill our definition of incident RA (eFigure 1 in the Supplement), leaving 8090 patients (5529 women and 2561 men; mean [SD] age, 58.3 [15.2] years) for further analysis (eTable 1 in the Supplement).

For each individual with RA, 10 reference participants from the Swedish Population Register were individually matched based on birth year (per calendar year), sex, and residential area. Participants had to be alive at the index date (ie, the date of diagnosis of RA for the individual with RA). In total, 80 782 reference participants were included. The methods of selection and the matching criteria were the same for the participants in the cohort study. Moreover, in the cohort study, participants with a history of AITD at diagnosis of RA, with a first thyroxine prescription before 2006 (see definition of incident AITD in the Occurrence of AITD subsection that follows), and with an index date after December 31, 2012, were excluded. In total, 6515 patients with RA (exposed cohort) and 58 228 reference participants (unexposed cohort) were included. They were followed up until the end of the study, death, emigration, or onset of AITD.

### Occurrence of AITD

In non–iodine-depleted areas, more than 90% of cases of hypothyroidism are considered to be autoimmune^18^ and are treated with long-term thyroxine substitution. After initial therapy for hyperthyroidism caused by autoimmune mechanisms (eg, Graves disease), most patients also need long-term thyroxine substitution for hypothyroidism. Other indications for thyroxine substitution are the adverse effects of iodine-containing drugs and the removal of the thyroid gland owing to cancer.^19^ There is no internationally accepted classification criteria defining AITD. However, after exclusion of nonautoimmune causes leading to hypothyroidism and thyroxine use, most patients treated with thyroxine substitution can be presumed to have AITD.^20^

For this study, we therefore defined AITD as a filling of a prescription of thyroid hormone substitution therapy, based on the Anatomical Therapeutic Chemical Classification codes for levothyroxine or liothyronine. By linkage to the PDR, we identified all participants treated with thyroxine substitution between 2005 and 2013. Through linkage to the Swedish Cancer Register and to the PDR, participants with a history of thyroid cancer or a prescription of iodine-containing drugs (lithium, amiodarone, or interferon-alfa) were identified and excluded (eFigure 1 in the Supplement).

We defined prevalent AITD as a history of at least 1 thyroxine prescription in the PDR from 2005 through 2013. Since the PDR was started in 2005, a prescription in this year could be either a first-ever prescription or a renewed prescription (eg, unknown onset of AITD). Incident AITD was therefore defined as a first thyroxine prescription in 2006 or later, and participants with a thyroxine prescription in 2005 were excluded (eFigure 1 in the Supplement). Autoimmune thyroid disease was further subgrouped as hyperthyroidism or hypothyroidism, based on linkage to the NPR. Hyperthyroidism was defined as a prescription for thyroxine and *ICD*-9 and *ICD-10* codes for Graves disease (see eAppendix 1 in the Supplement for Anatomical Therapeutic Chemical Classification and *ICD* codes used). Because most cases of hypothyroidism are treated in primary care visits not included in the NPR, hypothyroidism was defined as a prescription for thyroxine and an *ICD* code for hypothyroidism or the absence of an *ICD* code for hyperthyroidism.

Information on residential area, date of emigration, and date of death until December 31, 2013, was collected by linking to the Swedish Population Register and Cause of Death Register. Complete information on vital status such as sex and age was a prerequisite for inclusion into this type of register-based study. Less than 0.2% of individuals were excluded because of missing data on vital status. Registering a diagnosis code for RA is a prerequisite for inclusion into the SRQ, leading to no missing values on *ICD-10* code–based information on serostatus. The essentially complete information on date of death and emigration in the Swedish Population Register and the Cause of Death register and on date of prescriptions in the PDR allows for virtually no losses to follow-up.

### Statistical Analysis

Statistical analysis was performed from July 1, 2015, to June 30, 2017. To assess the prevalence and relative risk of prevalent AITD at diagnosis of RA, we used a matched case-control design. To calculate the relative risk of incident AITD before RA, we used a matched case-control design with the patients with RA as cases and their reference participants as controls. In this analysis, AITD occurring prior to diagnosis of RA was considered as exposure. The relative risk (odds ratio [OR]) of AITD was calculated in different strata based on the time before diagnosis of RA (<3 months, 3 to <12 months, 12 to <24 months, 24 to <60 months, and ≥60 months before diagnosis), using conditional logistic regression conditioned on the matching factors.

To calculate the relative risk of incident AITD after onset of RA, we used a matched cohort design with patients with RA as the exposed cohort, the reference participants as the unexposed cohort, and AITD as the outcome. In this cohort approach, participants with a history of AITD at the start of each follow-up interval and participants with an index date after December 31, 2012, were excluded to allow a minimum of 1 year of follow-up (until the end of the study, death, emigration, or onset of AITD). Relative risk was calculated as a hazard ratio with 95% CI using Cox proportional hazards regression, and it was calculated overall and by time since diagnosis of RA (0 to <3 months, 3 to <12 months, 12 to <24 months, 24 to <60 months, and ≥60 months).

All analyses of incident AITD were stratified by age, sex, and subtype of RA. In addition, separate analyses were performed for hyperthyroidism and hypothyroidism. We further explored the adjustment for other comorbidities that might be associated with detecting AITD (see eAppendix 2 in the Supplement for *ICD* codes used) and for the number of physician visits in the NPR in the 5 years prior to diagnosis of RA. All *P* values were from 2-sided tests and results were deemed statistically significant at *P* < .05. All analyses were performed using SAS, version 9.4 (SAS Institute Inc).

## Results

### Prevalence of AITD at Diagnosis of RA

At the time of diagnosis of RA, the prevalence of AITD was 10.3% (n = 832) among the 8066 patients with RA vs 7.1% (n = 5725) among the 80 350 matched population controls, corresponding to an OR of 1.5 (95% CI, 1.4-1.7). The relative risk of prevalent AITD was also assessed in relation to time before and after RA diagnosis (eTable 2 and eFigure 2 in the Supplement).

### Relative Risk of Incident AITD Before and After Diagnosis of RA, by Subtype of RA

The maximum observation time was 7 years before and 8 years after diagnosis of RA. During this period, we identified 374 cases of new-onset AITD in the group with RA vs 2587 in the general population comparator group (Table 1). The Figure describes the frequency of new-onset AITD in each time interval and the corresponding relative risk for incident AITD before, at, and after diagnosis of RA. The relative risk of AITD in the group with RA increased from no increase more than 5 years before diagnosis of RA, to a marginal increase 2 to 5 years before diagnosis, peaked the year before diagnosis, and then leveled off and turned into a decreased risk 5 or more years after diagnosis (Figure, Table 1, Table 2, and Table 3). Separate analyses stratified by age and subtype of RA revealed higher ORs for AITD among younger patients (16-49 years: OR, 2.8; 95% CI, 2.2-3.5) and those with seropositive RA (OR, 1.8; 95% CI, 1.5-2.1) (Table 1). When incident AITD was analyzed separately for seropositive and seronegative RA, the pattern of relative risk was, for the most part, similar to that observed in the whole group with RA (eTable 3 and eTable 4 in the Supplement).

**Table 1.** Relative Risk of Incident AITD Before and Then After the Diagnosis of RA Among 7489 Patients With RA in the SRQ Compared With 70 965 Matched Population Controls^a^

Characteristic	Patients with RA, No.	Population Controls, No.	OR or HR (95% CI)^b^
Overall before RA diagnosis	255	1442	1.7 (1.5-2.0)^c^
Sex			
Female	219	1226	1.7 (1.5-2.0)^c^
Male	36	216	1.6 (1.1-2.3)^c^
RF status among cases			
Positive	164	875	1.8 (1.5-2.1)^c^
Negative	81	501	1.5 (1.2-1.9)^c^
NA	10	66	1.5 (0.7-2.9)^c^
Time between AITD and RA diagnosis, mo			
0 to <3	47	84	5.3 (3.7-7.6)^c^
3 to <12	49	233	2.0 (1.5-2.7)^c^
12 to <24	42	284	1.4 (1.0-1.9)^c^
24 to <60	96	627	1.5 (1.2-1.8)^c^
≥60	21	214	0.9 (0.6-1.4)^c^
Age at inclusion in SRQ, y			
16 to 49	86	315	2.8 (2.2-3.5)^c^
50 to 74	135	893	1.4 (1.2-1.7)^c^
>74	34	234	1.3 (0.9-1.9)^c^
Overall after RA diagnosis	119	1145	0.9 (0.8-1.1)^d^
Time since RA diagnosis, mo			
0 to <3	15	62	2.1 (1.2-3.7)^d^
3 to <12	26	205	1.1 (0.7-1.7)^d^
12 to <24	29	249	1.1 (0.7-1.6)^d^
24 to <60	40	485	0.7 (0.5-1.0)^d^
≥60	9	144	0.6 (0.3-1.1)^d^

Abbreviations: AITD, autoimmune thyroid disease; HR, hazard ratio; NA, not available; OR, odds ratio; RA, rheumatoid arthritis; RF, rheumatoid factor; SRQ, Swedish Rheumatology Quality Register.

^a^ Participants with thyroxine treatment and treatment with iodine-containing drugs or a history of thyroid cancer were excluded.

^b^ Adjusted for matching factors; age, sex, and residential area.

^c^ Odds ratio assessed by conditional logistic regression.

^d^ Hazard ratio assessed by Cox proportional hazards regression.

**Figure.**
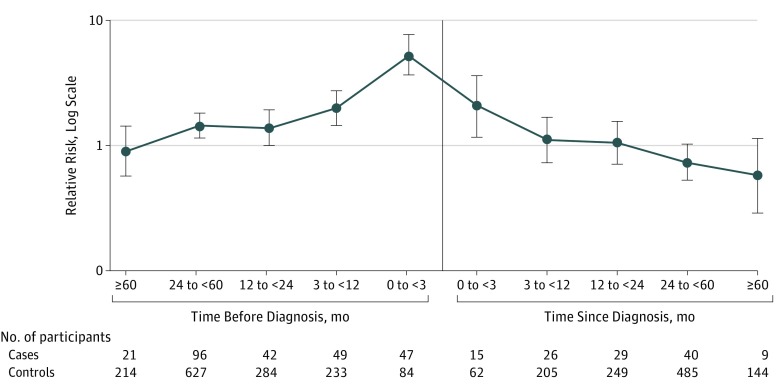
Relative Risk of Incident Autoimmune Thyroid Disease Before and After Diagnosis of Rheumatoid Arthritis The error bars indicate 95% CIs.

**Table 2.** Relative Risk of Incident Hypothyroidism Before and Then After the Diagnosis of RA Among 7471 Patients With RA in the SRQ Compared With 70 653 Matched Controls^a^

Characteristic	Patients With RA, No.	Population Controls, No.	OR or HR (95% CI)^b^
Overall before RA diagnosis	237	1290	1.7 (1.5-2.0)^c^
Sex			
Female	204	1083	1.8 (1.5-2.1)^c^
Male	33	207	1.5 (1.0-2.2)^c^
RF status among cases			
Positive	152	784	1.8 (1.5-2.2)^c^
Negative	75	447	1.6 (1.2-2.0)^c^
NA	10	59	1.6 (0.8-3.2)^c^
Time between hypothyroidism and RA diagnosis, mo			
0 to <3	43	79	5.1 (3.6-7.5)^c^
3 to <12	46	207	2.1 (1.5-2.9)^c^
12 to <24	40	252	1.5 (1.1-2.1)^c^
24 to <60	89	557	1.5 (1.2-1.9)^c^
≥60	19	195	0.9 (0.6-1.4)^c^
Age at inclusion in SRQ, y			
16 to 49	84	278	3.1 (2.4-3.9)^c^
50 to 74	120	798	1.4 (1.2-1.7)^c^
>74	33	214	1.4 (1.0-2.0)^c^
Overall after RA diagnosis	117	1044	1.0 (0.8-1.2)^d^
Time since RA diagnosis, mo			
0 to <3	15	55	2.3 (1.3-4.2)^d^
3 to <12	25	181	1.2 (0.8-1.8)^d^
12 to <24	29	236	1.1 (0.8-1.6)^d^
24 to <60	39	442	0.8 (0.6-1.1)^d^
≥60	9	130	0.6 (0.3-1.2)^d^

Abbreviations: HR, hazard ratio; NA, not available; OR, odds ratio; RA, rheumatoid arthritis; RF, rheumatoid factor; SRQ, Swedish Rheumatology Quality Register.

^a^ Number of cases and controls at index date.

^b^ Adjusted for matching factors: age, sex, and residential area.

^c^ Odds ratio assessed by conditional logistic regression.

^d^ Hazard ratio assessed by Cox proportional hazards regression.

**Table 3.** Relative Risk of Incident Hyperthyroidism Before and Then After the Diagnosis of RA Among 7252 Patients With RA in the SRQ Compared With 67 498 Matched Controls^a^

Characteristic	Patients With RA, No.	Population Controls, No.	OR or HR (95% CI)^b^
Overall before RA diagnosis	18	145	1.1 (0.7-1.8)^c^
Sex			
Female	15	136	1.0 (0.6-1.7)^c^
Male	3	9	3.2 (0.9-11.8)^c^
RF status among cases			
Positive	12	87	1.3 (0.7-2.2)^c^
Negative	6	51	1.0 (0.4-2.4)^c^
NA	0	7	NA
Time between hyperthyroidism and RA diagnosis, mo			
0 to <3	4	4	9.0 (2.2-35.9)^c^
3 to <12	3	25	1.1 (0.3-3.7)^c^
12 to <24	2	31	0.6 (0.1-2.4)^c^
24 to <60	7	66	1.0 (0.4-2.1)^c^
≥60	2	19	1.0 (0.2-4.1)^c^
Age at inclusion in SRQ, y			
16 to 49	2	37	0.5 (0.1-2.2)^c^
50 to 74	15	88	1.6 (0.9-2.7)^c^
>74	1	20	0.4 (0.1-2.9)^c^
Overall after RA diagnosis	2	98	0.2 (0.05-0.8)^d^
Time since RA diagnosis, mo			
0 to <3	0	7	NA
3 to <12	1	24	0.4 (0.05-2.8)^d^
12 to <24	0	13	NA
24 to <60	1	42	0.2 (0.03-1.6)^d^
≥60	0	12	NA

Abbreviations: HR, hazard ratio; NA, not available; OR, odds ratio; RA, rheumatoid arthritis; RF, rheumatoid factor; SRQ, Swedish Rheumatology Quality Register.

^a^ Number of cases and controls at index date.

^b^ Adjusted for matching factors: age, sex, and residential area.

^c^ Odds ratio assessed by conditional logistic regression.

^d^ Hazard ratio assessed by Cox proportional hazards regression.

### Relative Risk of Incident AITD Before and After Diagnosis of RA, by Subtype of AITD

When the analysis of AITD was restricted to hypothyroidism (94.7% [354 of 374] of all incident cases of AITD in the group with RA and 90.2% [2334 of 2587] of all incident cases of AITD in the population comparator group), the pattern of relative risk was similar to that observed for incident AITD overall (Table 2). At the time of diagnosis of RA, 18 of 7252 patients with RA (0.2%) and 145 of the 67 498 population controls (0.2%) had a history of hyperthyroidism, corresponding to no overall increase in risk among the patients with RA (adjusted OR, 1.1; 95% CI, 0.7-1.8). By contrast, when different strata of time were assessed separately, we observed a substantial increase in the relative risk of hyperthyroidism 0 to 3 months before the diagnosis of RA (OR, 9.0; 95% CI, 2.2-35.9), followed by a decreased risk after diagnosis (adjusted hazard ratio 0.2; 95% CI, 0.05-0.8) (Table 3). Adjustment for comorbidities that might be related to the likelihood of detecting AITD and the number of physician visits (eTable 5 in the Supplement) had little association with the relative risk estimates (eTable 6 in the Supplement).

## Discussion

The results of this study extend previous findings of an increased occurrence of AITD among patients with RA by demonstrating that much of the increased risk of AITD by the time of diagnosis of RA develops during the last few years before diagnosis and that, thereafter, the incidence of AITD is no longer elevated but decreased.

### Results in Context

The prevalence of AITD among patients with RA in our study, approximately 10% at diagnosis, is in line with previous studies.^6,8,13^ The criteria for AITD, however, differ between studies, which, apart from the timing of the measurement, may be one explanation for the wide range of previously reported estimates of prevalence.^14,21^ The timing of risk development seen in our study with an increased risk by the time of diagnosis of RA, is consistent with previous findings from a case-control study based on self-reported AITD and treatment.^6^

### Potential Mechanisms

The co-occurrence of RA and AITD may be due to several factors. The number of identified, shared susceptibility genes for AITD and RA is increasing, and multiple additional loci have been suggested.^22,23^ It remains unclear, however, whether there is an increased prevalence of antithyroid antibodies among patients with RA; the findings^24,25^ have been somewhat contradictory, including the indication that antithyroid autoantibodies may become negative over time.^26^

The timing of the risk development of AITD in relation to the onset of RA may have additional explanations. During a period of time around the diagnosis of RA, the diagnostic intensity is likely increased. Considering the often-insidious onset of hypothyroidism, an earlier detection (lead-time bias) would manifest itself as an increased incidence followed by a reciprocal decrease. Because we included only patients with RA with a self-reported symptom duration of less than 12 months before diagnosis, any increased diagnostic intensity should be limited to this period, yet we observed an increased incidence several years earlier. In our study, even the extreme assumption that all new-onset AITD up to 5 years after diagnosis of RA would be detected during the year before diagnosis of RA (lead-time bias) would only explain two-thirds of the excess cases in the incidence peak around diagnosis. In addition, a surveillance bias would presumably also continue sometime after diagnosis of RA, yet the relative incidence of AITD decreased rapidly during this period. Lead-time bias would not explain the previously reported increased occurrence of thyroid antibodies in patients with RA.^7,8,12,13,14^ Besides lead time, the observed peak in risk of AITD shortly prior to diagnosis of RA may also reflect a possible critical causal link between the 2 autoimmune conditions, with one (AITD) triggering the clinical onset of the other (RA) in genetically predisposed individuals. Such a mechanism may explain the higher incidence seen among seropositive patients compared with seronegative patients, for whom there should not be any difference in diagnostic intensity or lead-time bias.

It remains an open question whether a potential reciprocal deficit after a period of increased diagnostic surveillance around the diagnosis of RA may explain the persistent decrease in new-onset AITD years after the onset of RA. An alternative explanation includes a protective effect of antirheumatic therapies on the development of AITD. A preclinical study of mice found that anti–tumor necrosis factor treatment reduced the expression of proinflammatory cytokines in the thyroid gland, leading to less inflammation,^27^ and 2 clinical studies reported improved thyroid function in patients with RA and hypothyroidism who received anti–tumor necrosis factor treatment.^28,29^

Although the pattern of risk before diagnosis of RA was similar for hyperthyroidism and hypothyroidism, the decrease in risk after diagnosis of RA was more pronounced for hyperthyroidism. The differences in autoimmune mechanisms between the 2 subsets might be 1 explanation. However, the numbers of patients with new-onset hyperthyroidism were limited, and potential differences in risk need to be further investigated.

When adjusting for comorbidities, the pattern of relative risk remained similar to that observed for incident AITD overall (eTable 6 in the Supplement). Adjustment for these comorbidities separately resulted in a slightly attenuated association between RA and AITD for type 1 diabetes, malaise and fatigue, myalgia, osteoporosis, and pregnancy-related outcomes. By contrast, when adjusting for depression, dementia, and hyperparathyroidism, we found that the association was slightly enhanced. When adjusting for the number of physician visits in the NPR prior to a diagnosis of RA, we found that the association was slightly attenuated (eTable 6 in the Supplement).

### Strengths and Limitations

Our study has several strengths. By using an inception cohort retrieved from high-coverage registers with information on dates of onset of symptoms and diagnosis of RA, we could specifically assess the risk for AITD by the time before and after diagnosis of RA. The large size of our cohort of patients with RA provided the possibility to assess risk in time strata and subgroups. We used a population-based design and could sample controls from the same study base as the cases, minimizing the risk of selection bias. Furthermore, we used prospectively recorded information from the nationwide and virtually complete drug register for information on thyroxine substitution, independently of RA status, thus minimizing the risk of bias due to selective recall and outcome ascertainment.

Our study has some limitations. The study population was conditioned on being alive at the time of the diagnosis of RA. If AITD were linked to a markedly different short-term survival in cases of RA, it could introduce a selection bias in the assessment of risk prior to the diagnosis of RA. We used the date of the first prescription of thyroxine as a proxy for clinical onset of AITD and the date of the first diagnosis of RA as a proxy for clinical onset of RA. However, the pathogenic mechanisms underlying RA and AITD are likely to have started months or even years earlier.

The levels of autoantibodies against thyroid structures are reported to be around 12% in the general population, but the prevalence of clinically overt AITD, however, is lower.^30^ We did not have information on levels of autoantibodies or thyroid-stimulating hormones in our study. Thyroxine substitution has been considered an adequate proxy for capturing clinically overt AITD, after exclusion of nonautoimmune indications.^6,20^ We found this proxy to be appropriate for our study because we wanted to investigate the temporal pattern for clinically overt AITD in RA. Although it cannot be ruled out that some participants may still have a nonautoimmune indication for thyroxine treatment, the introduction of a potential misclassification of AITD should not depend on the presence of RA and thus should not affect the associations observed. Apart from the matching factors and comorbidities, we did not have information about potential confounders of the association between RA and AITD, such as body mass index and smoking. In a previous case-control study, adjustment for smoking did not explain the increased prevalence of AITD.^6^ Uncontrolled confounding cannot, however, be formally discounted.

### Clinical Implications

Our results highlight that patients with AITD who have joint symptoms should be evaluated for a potential new-onset RA. Conversely, patients with RA may be reassured that their risk of also developing AITD is not increased.

## Conclusions

Rheumatoid arthritis is associated with an increased occurrence of AITD as a result of an increased risk of development of AITD a few years before diagnosis of RA. On the other hand, starting from diagnosis of RA, the risk of developing new-onset AITD is gradually reduced. The risk of AITD varied between its subsets, hypothyroidism and hyperthyroidism, and with age, sex, and RA subgroup, but the temporal pattern of risk was similar across the patient subgroups. Why the risk of developing AITD seems to decrease after diagnosis of RA and whether this decrease in risk represents a protective effect of immunomodulatory therapies need to be further investigated.

## Notes

Supplement. **eFigure 1.** Flowchart of RA Cases, General Population Controls and Incident AITD **eFigure 2.** Cumulative Prevalence and Relative Risk of AITD in Relation to RA Diagnosis **eAppendix 1.** ATC and *ICD* Codes **eAppendix 2.** *ICD* Codes for Comorbidities **eTable 1.** Baseline Characteristics of 8090 RA Patients Identified in the Swedish Rheumatology Quality Register (SRQ), 2006-2013 **eTable 2.** Cumulative Prevalence and Relative Risk of Prevalent AITD in Relation to RA Diagnosis **eTable 3.** Relative Risk of Incident AITD Before and After the Diagnosis of Seropositive RA in 4785 Patients With RA Compared With 45 342 Matched Population Controls **eTable 4.** Relative Risk of Incident AITD Before and After the Diagnosis of Seronegative or Unspecified RA in 2704 Patients With RA Compared With 25 623 Matched Population Controls **eTable 5.** Number of Physician Visits in the NPR the Last 5 Years Before RA Diagnosis Among 7489 Patients With RA Compared With 70 965 Matched Population Controls **eTable 6.** Relative Risk of Incident AITD Before and Then After the Diagnosis of RA in 7489 Patients With RA Compared With 70 965 Matched Population Controls
