# Supplementary material for: Risk of Thyroxine-Treated Autoimmune Thyroid Disease Associated With Disease Onset in Patients With Rheumatoid Arthritis
Source: JAMA Netw Open. 2018 Oct 19;1(6):e183567. doi: 10.1001/jamanetworkopen.2018.3567 (PMC6324433; doi:10.1001/jamanetworkopen.2018.3567)
Supplement: Supplement. — eFigure 1. Flowchart of RA Cases, General Population Controls and Incident AITD eFigure 2. Cumulative Prevalence and Relative Risk of AITD in Relation to RA Diagnosis eAppendix 1. ATC and ICD Codes eAppendix 2. ICD Codes for Comorbidities eTable 1. Baseline Characteristics of 8090 RA Patients Identified in the Swedish Rheumatology Quality Register (SRQ), 2006-2013 eTable 2. Cumulative Prevalence and Relative Risk of Prevalent AITD in Relation to RA Diagnosis eTable 3. Relative Risk of Incident AITD Before and After the Diagnosis of Seropositive RA in 4785 Patients With RA Compared With 45 342 Matched Population Controls eTable 4. Relative Risk of Incident AITD Before and After the Diagnosis of Seronegative or Unspecified RA in 2704 Patients With RA Compared With 25 623 Matched Population Controls eTable 5. Number of Physician Visits in the NPR the Last 5 Years Before RA Diagnosis Among 7489 Patients With RA Compared With 70 965 Matched Population Controls eTable 6. Relative Risk of Incident AITD Before and Then After the Diagnosis of RA in 7489 Patients With RA Compared With 70 965 Matched Population Controls [file jamanetwopen-1-e183567-s001.pdf]

Supplementary Online Content

Waldenlind K, Saevarsdottir S, Bengtsson C, Askling J. Risk of thyroxine-treated autoimmune thyroid disease associated with disease onset in patients with rheumatoid arthritis. *JAMA Netw Open.* 2018;1(6):e183567. doi:10.1001/jamanetworkopen.2018.3567

eFigure 1. Flowchart of RA Cases, General Population Controls and Incident AITD

eFigure 2. Cumulative Prevalence and Relative Risk of AITD in Relation to RA Diagnosis

eAppendix 1. ATC and *ICD* Codes

eAppendix 2. *ICD* Codes for Comorbidities

eTable 1. Baseline Characteristics of 8090 RA Patients Identified in the Swedish Rheumatology Quality Register (SRQ), 2006-2013

eTable 2. Cumulative Prevalence and Relative Risk of Prevalent AITD in Relation to RA Diagnosis

eTable 3. Relative Risk of Incident AITD Before and After the Diagnosis of Seropositive RA in 4785 Patients With RA Compared With 45 342 Matched Population Controls

eTable 4. Relative Risk of Incident AITD Before and After the Diagnosis of Seronegative or Unspecified RA in 2704 Patients With RA Compared With 25 623 Matched Population Controls

eTable 5. Number of Physician Visits in the NPR the Last 5 Years Before RA Diagnosis Among 7489 Patients With RA Compared With 70 965 Matched Population Controls

eTable 6. Relative Risk of Incident AITD Before and Then After the Diagnosis of RA in 7489 Patients With RA Compared With 70 965 Matched Population Controls

This supplementary material has been provided by the authors to give readers additional information about their work.

© 2018 Waldenlind K et al. *JAMA Network Open.*

eFigure 1. Flowchart of RA Cases, General Population Controls and Incident AITD

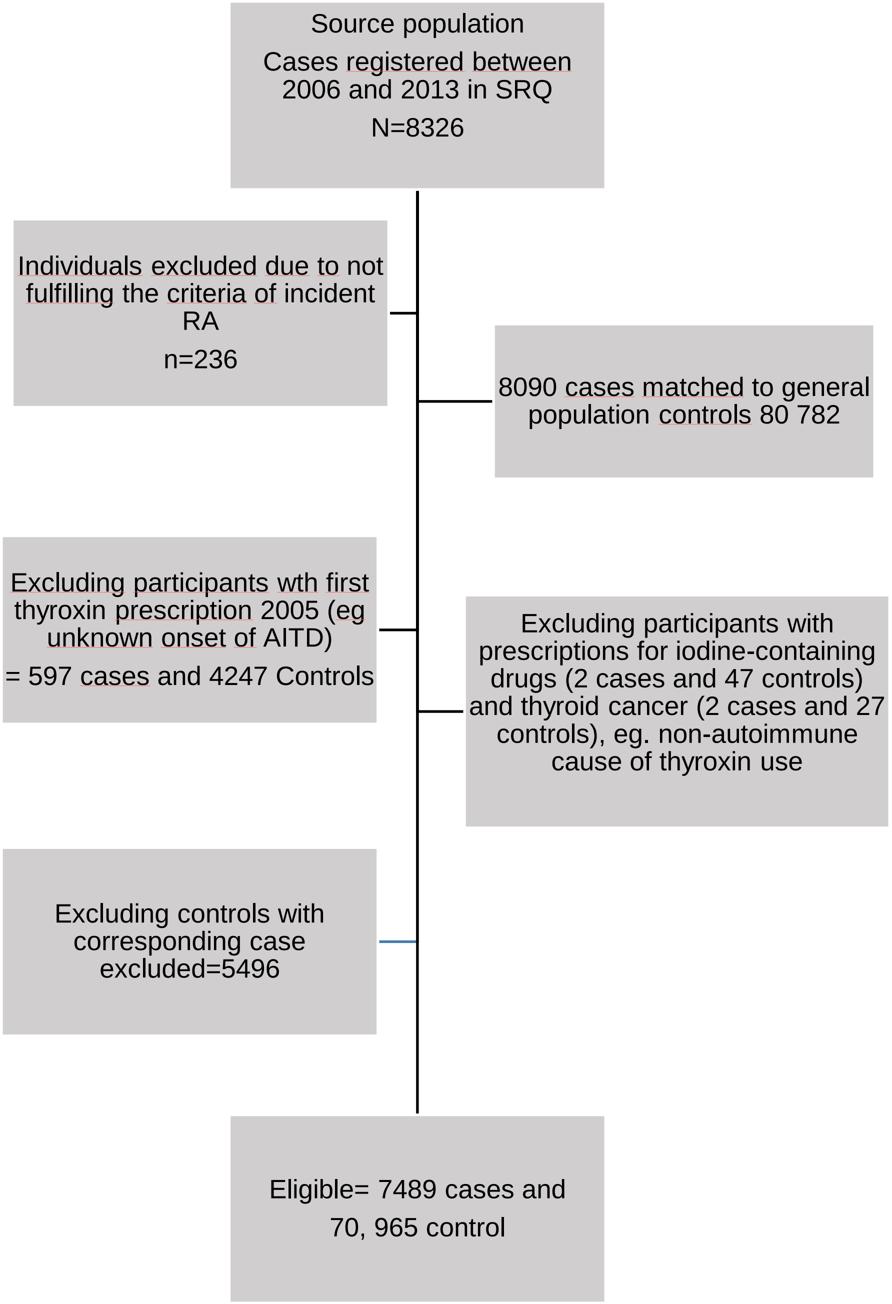

© 2018 Waldenlind K et al. *JAMA Network Open.*

eFigure 2. Cumulative Prevalence and Relative Risk of AITD in Relation to RA Diagnosis

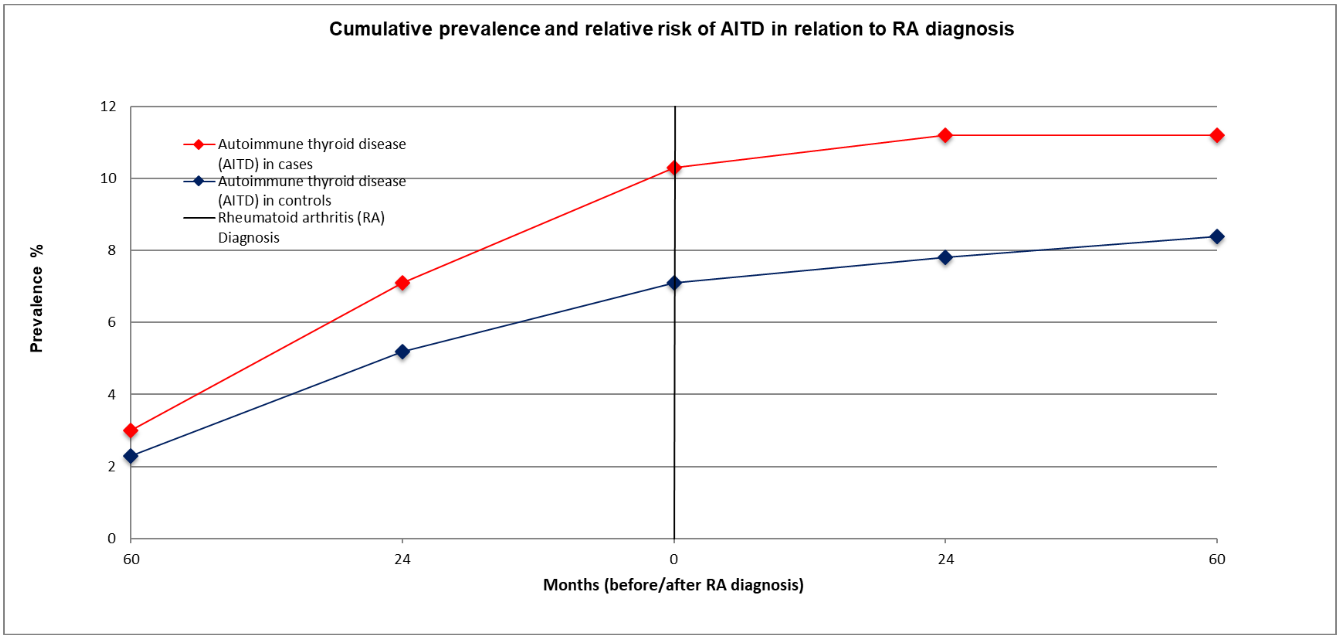

© 2018 Waldenlind K et al. *JAMA Network Open.*

eAppendix 1. ATC and *ICD* Codes

ATC codes in the Swedish prescribed drug register:

H03AA01= levothyroxine, T4 (levaxin+euthyrox), H03AA02= liothyronin, T3 (liothyronin)

Excluding participants with prescriptions for iodine-containing drugs in the Swedish prescribed drug register: ATC-code: C01BD01 (Amiodarone), ATC-code: N05AN01 (Litium), (ATC-code:L03AB01/L03AB04/L03AB05 (Interferon-alfa/alfa 2a/alfa 2b).

***ICD*-codes in the Swedish cancer register**:

Excluding participants with diagnosis codes for thyroid cancer in the Swedish cancer register *icd7*=194 or *icd9*=193 or *icd10*=c73

***ICD*-codes in the Swedish National Patient Register (NPR) for hypothyreodism and hyperthyreodism (*ICD 9* and *ICD 10*):**

Hypothyroidism: E038, E039, E063, 244x, 245c

Hyperthyroidism: E050, E051, E052, E053, E058, E059, 242, 242A, 242B, 242D, 242E

Postpartum thyroiditis=O905

© 2018 Waldenlind K et al. *JAMA Network Open.*

eAppendix 2. *ICD* Codes for Comorbidities

Depressive episode: F32.0, F32.1, F32.2, F32.3, F32.8, F32.9, F33.0, F33.1, F33.2, F33.3, F33.4, F33.8, F33.9

Insulin-dependent diabetes mellitus: E10.1, E10.1A, E10.1B, E10.1D, E10.1X, E10.5A, E10.5B, E10.5W, E10.5X, E10.5, E10.7, E10.9

Osteoporosis: M80.0, M80.0A, M80.0B, M80.0C, M80.0E, M80.0F, M80.0G, M80.0H, M80.0J, M80.0K, M80.5, M80.8, M81.0, M81.5, M81.8, M81.9, M82.1, M82.8

Dementia: F00.1, F00.2, F00.9, F01.2, F01.3, F01.9, F02.8, F03.9, F06.7

Primary hyperparathyroidism: E21.0, E21.3, E21.1

Malaise and fatigue: R53.9

Burn out (state): Z73.0

Myalgia: M79.1

Pregnancy related conditions: O24.4, O26.8, O21.1, Z34.9, O99.8, O21.9, O26.9

© 2018 Waldenlind K et al. *JAMA Network Open.*

eTable 1. Baseline Characteristics of 8090 RA Patients Identified in the Swedish Rheumatology Quality Register (SRQ), 2006-2013[a]

Characteristic	RA patients
Sex	
women	5529 (68.3)
men	2561 (31.7)
RA diagnosis[b]	
seropositive RA	5195 (64.2)
seronegative RA	2612 (32.3)
unspecified RA	283 (3.5)
Year of inclusion in SRQ	
2006	883 (10.9)
2007	953 (11.8)
2008	1003 (12.4)
2009	1173 (14.5)
2010	1103 (13.6)
2011	1156 (14.3)
2012	1228 (15.2)
2013[c]	591 (7.3)
Age at inclusion in SRQ/indexdate (mean+/- SD years)	58.3 (15.2)

[a] Values are the numbers in groups (%).

[b] According to the *ICD 10* registered,

assessed at the time of diagnosis of RA, i.e., inclusion in the SRQ.

[c] Until June 30, 2013.

© 2018 Waldenlind K et al. *JAMA Network Open.*

eTable 2. Cumulative Prevalence and Relative Risk of Prevalent AITD in Relation to RA Diagnosis

	**No. RA cases**	**No. Population controls**	**OR (95% CI)**
5 years or more *before* RA diagnosis[a]	239 (3.0%)	1842 (2.3%)	1.3 (1.2-1.5)
2 years or more *before* RA diagnosis	572 (7.1%)	4193 (5.2%)	1.4 (1.3-1.5)
At RA diagnosis	832 (10.3%)	5725 (7.1%)	1.5 (1.4-1.7)
2 years *after* RA diagnosis[b]	671 (11.2%)	4299 (7.7%)	1.5 (1.4-1.7)
5 years *after* RA diagnosis[c]	293 (11.2%)	1950 (8.2%)	1.4 (1.2-1.6)

Values are the numbers in groups;
Abbrevation: RA= rheumatoid arthritis; AITD= autoimmune thyroid disease;
OR= odds ratio;
95% CI= 95% confidence interval (assessed by conditional logistic regression)
[a]Cases n=8066, controls n= 80 350, (participants excluded with non-autoimmune reason for thyroxin treatment)
[b]cases=5975, controls 56 015, (participants excluded with 2 years follow up date after 2013.12.31, date of death or emigration before 2 years follow-up date, or non-autoimmune reason for thyroxin treatment)
[c]cases n=2618, controls n=23 655, (participants excluded with 5 years follow up date after 2013.12.31, date of death or emigration before 5 years follow-up date, or non-autoimmune reason for thyroxin treatment)

© 2018 Waldenlind K et al. *JAMA Network Open.*

eTable 3. Relative Risk of Incident AITD Before and After the Diagnosis of Seropositive RA in 4785 Patients With RA Compared With 45 342 Matched Population Controls[a]

	**No. RA cases**	**No. Population controls**	**OR (95% CI)[b]**
**Overall before RA diagnosis**	164	875	1.8 (CI 1.5-2.1)
Time between AITD and RA diagnosis			
0-<3 months	30	53	5.5 (3.5-8.6)
3-<12 months	30	146	2.0 (1.3-2.9)
12-<24 months	26	169	1.4 (1.0-2.2)
24-<60 months	64	367	1.7 (1.3-2.2)
>60 months	14	140	0.9 (0.5-1.6)
			**HR (95% CI)[b]**
**Overall after RA diagnosis**	76	738	0.9 (0.7-1.2)
Time since RA diagnosis			
0-<3 months	13	39	2.9 (1.5-5.4)
3-<12 months	17	123	1.2 (0.7-2.0)
12-<24 months	18	161	1.0 (0.6-1.7)
24-<60 month	23	320	0.6 (0.4-1.0)
>60 months	5	95	0.5 (0.2-1.2)

Values are the numbers in groups;

Abbrevation: RA= rheumatoid arthritis; AITD= autoimmune thyroid disease;

OR= odds ratio (assessed by conditional logistic regression); HR= hazard ratio (assessed by cox regression);

95% CI= 95% confidence interval;

[a]Participants with thyroxin treatment and treatment with iodine-containing drugs or a history of thyroid cancer excluded;

[b]Adjusted for matching factors; age, sex, residential area

© 2018 Waldenlind K et al. *JAMA Network Open.*

eTable 4. Relative Risk of Incident AITD Before and After the Diagnosis of Seronegative or Unspecified RA in 2704 Patients With RA Compared With 25 623 Matched Population Controls[a]

	No. RA cases	No. Population controls	OR (95% CI)[b]
**Overall before RA diagnosis**	91	567	1.5 (1.2-1.9)
Time between AITD and RA diagnosis			
0-<3 months	17	31	5.1 (2.8-9.2)
3-<12 months	19	87	2.1 (1.3-3.4)
12-<24 months	16	115	1.3 (0.8-2.2)
24-<60 months	32	260	1.2 (0.8-1.7)
>60 months	7	74	0.9 (0.4-1.9)
			**HR (95% CI)[b]**
**Overall after RA diagnosis**	43	407	0.9 (0.7-1.3)
Time since RA diagnosis			
0-<3 months	2	23	0.7 (0.2-3.1)
3-<12 months	9	82	1.0 (0.5-1.9)
12-<24 months	11	88	1.1 (0.6-2.1)
24-<60 month	17	165	0.9 (0.6-1.5)
>60 months	4	49	0.7 (0.3-2.0)

Values are the numbers in groups;

Abbrevation: RA= rheumatoid arthritis; AITD= autoimmune thyroid disease;

OR= odds ratio (assessed by conditional logistic regression); HR= hazard ratio (assessed by cox regression);

95% CI= 95% confidence interval;

[a]Participants with thyroxin treatment and treatment with iodine-containing drugs or a history of thyroid cancer excluded;

[b]Adjusted for matching factors; age, sex, residential area

© 2018 Waldenlind K et al. *JAMA Network Open.*

eTable 5. Number of Physician Visits in the NPR the Last 5 Years Before RA Diagnosis Among 7489 Patients With RA Compared With 70 965 Matched Population Controls[a]

	**RA cases**		**Population controls**	
	Median (IQRs)	Mean (SD)	Median (IQRs)	Mean (SD)
**Overall last 5 years before RA diagnosis**	5.0 (8.0)	7.7 (10.9)	3.0 (6.0)	5.7 (10.6)
Time intervals				
0-<3 months	1.0 (1.0)	1.7 (1.4)	0.0 (0.0)	0.3 (1.1)
3-<12 months	0.0 (2.0)	1.4 (2.8)	0.0 (1.0)	1.0 (2.6)
12-<24 months	0.0 (2.0)	1.4 (3.0)	0.0 (1.0)	1.2 (3.0)
24-<60 months	1.0 (4.0)	3.3 (6.6)	1.0 (4.0)	3.1 (6.4)

Abbrevation: RA= rheumatoid arthritis; AITD= autoimmune thyroid disease; NPR=National Patient Register

[a]Participants with thyroxin treatment and treatment with iodine-containing drugs or a history of thyroid cancer excluded;

© 2018 Waldenlind K et al. *JAMA Network Open.*

eTable 6. Relative Risk of Incident AITD Before and Then After the Diagnosis of RA in 7489 Patients With RA[a] Compared With 70 965 Matched Population Controls[a]

	RA Cases	Controls	OR (95% CI)[b]	OR (95% CI)[c]	OR (95% CI)[d]	OR (95% CI)[e]
**Overall before RA**	255	1442	1.7 (1.5-1.9)	1.7 (1.5-1.9)	1.6 (1.4-1.9)	1.6 (1.4-1.9)
Sex						
women	219	1226	1.7 (1.5-2.0)	1.7 (1.4-1.9)	1.6 (1.4-1.9)	1.6 (1.4-1.9)
men	36	216	1.6 (1.1-2.3)	1.6 (1.1-2.3)	1.5 (1.1-2.2)	1.6 (1.1-2.3)
RF status among cases						
Positive	164	875	1.8 (1.5-2.1)	1.8 (1.5-2.1)	1.7 (1.5-2.1)	1.8 (1.5-2.1)
Negative	81	501	1.5 (1.2-1.9)	1.5 (1.2-1.9)	1.5 (1.2-1.9)	1.5 (1.1-1.9)
NA	10	66	1.5 (0.7-2.9)	1.5 (0.8-3.0)	1.4 (0.7-2.8)	1.5 (0.7-2.9)
Time between AITD and RA						
0-<3 months	47	84	5.3 (3.7-7.6)	5.4 (3.7-7.7)	5.3 (3.7-7.6)	5.3 (3.7-7.7)
3-<12 months	49	233	2.0 (1.5-2.7)	2.0 (1.5-2.8)	2.0 (1.5-2.7)	2.0 (1.5-2.8)
12-<24 months	42	284	1.4 (1.0-1.9)	1.4 (1.0-1.9)	1.3 (1.0-1.8)	1.3 (1.0-1.9)
24-<60 months	96	627	1.5 (1.2-1.8)	1.4 (1.1-1.8)	1.4 (1.1-1.7)	1.4 (1.1-1.7)
>60 months	21	214	0.9 (0.6-1.4)	0.9 (0.6-1.4)	0.9 (0.5-1.3)	0.9 (0.6-1.4)
Age at inclusion in SRQ						
16-49 years	86	315	2.8 (2.2-3.5)	2.7 (2.2-3.5)	2.5 (1.9-3.2)	2.5 (2.0-3.2)
50-74 years	135	893	1.4 (1.2-1.7)	1.4 (1.2-1.7)	1.4 (1.1-1.7)	1.4 (1.1-1.7)
>74 years	34	234	1.3 (0.9-1.9)	1.3 (0.9-1.9)	1.3 (0.9-1.9)	1.3 (0.9-1.9)
			**HR (95% CI)[b]**	**HR (95% CI)[b]**	**HR (95% CI)[b]**	**HR (95% CI)[b]**
**Overall after RA**	119	1145	0.9 (0.8-1.1)	0.9 (0.8-1.1)	0.9 (0.8-1.1)	0.9 (0.8-1.1)
Time since RA diagnosis						
0-<3 months	15	62	2.1 (1.2-3.7)	2.2 (1.2-3.8)	2.1 (1.2-3.6)	2.1 (1.2-3.7)
3-<12 months	26	205	1.1 (0.7-1.7)	1.1 (0.7-1.7)	1.1 (0.7-1.7)	1.1 (0.7-1.7)
12-<24 months	29	249	1.1 (0.7-1.6)	1.1 (0.7-1.5)	1.0 (0.7-1.5)	1.0 (0.7-1.5)
24-<60 month	40	485	0.7 (0.5-1.0)	0.7 (0.5-1.0)	0.7 (0.5-1.0)	0.7 (0.5-1.0)
>60 months	9	144	0.6 (0.3-1.1)	0.6 (0.3-1.1)	0.6 (0.3-1.1)	0.6 (0.3-1.1)

Values are the numbers in groups;

Abbrevation: RA= rheumatoid arthritis; AITD= autoimmune thyroid disease;

OR= odds ratio (assessed by conditional logistic regression); HR= hazard ratio (assessed by cox regression); 95% CI= 95% confidence interval;

[a] Participants with thyroxin treatment and treatment with iodine-containing drugs or a history of

thyroid cancer excluded;

[b] Adjusted for matching factors; age, sex, residential area

[c] Adjusted for matching factors; age, sex, residential area and comorbidities (depressive episode, insulin-dependent diabetes mellitus, osteoporosis, dementia, primary hyperparathyroidism, malaise and fatigue, burn out (state), myalgia, pregnancy related conditions (not adjusted for men))

[d] Adjusted for matching factors; age, sex, residential area and number of visits last 5 years prior to RA-diagnosis

[e] Adjusted for matching factors; age, sex, residential area, comorbidities and number of visits last 5 years prior to RA-diagnosis

© 2018 Waldenlind K et al. *JAMA Network Open.*
